# An Emerging Role of PRRT2 in Regulating Growth Cone Morphology

**DOI:** 10.3390/cells10102666

**Published:** 2021-10-05

**Authors:** Elisa Savino, Fabrizia Claudia Guarnieri, Jin-Wu Tsai, Anna Corradi, Fabio Benfenati, Flavia Valtorta

**Affiliations:** 1 Division of Neuroscience, IRCCS San Raffaele Scientific Institute, Via Olgettina 60, 20132 Milan, Italy; savino.elisa@hsr.it (E.S.); fabrizia.guarnieri@in.cnr.it (F.C.G.); 2 School of Medicine, Vita-Salute San Raffaele University, Via Olgettina 58, 20132 Milan, Italy; 3 Institute of Brain Science, School of Medicine, National Yang Ming Chiao Tung University, Taipei 11221, Taiwan; tsaijw@ym.edu.tw; 4 Center for Synaptic Neuroscience and Technology, Istituto Italiano di Tecnologia, Largo Rosanna Benzi 10, 16132 Genova, Italy; acorradi@unige.it (A.C.); Fabio.Benfenati@iit.it (F.B.); 5 IRCCS Ospedale Policlinico San Martino, Largo Rosanna Benzi 10, 16132 Genova, Italy

**Keywords:** PRRT2, actin, growth cone

## Abstract

Mutations in the PRRT2 gene are the main cause for a group of paroxysmal neurological diseases including paroxysmal kinesigenic dyskinesia, episodic ataxia, benign familial infantile seizures, and hemiplegic migraine. In the mature central nervous system, the protein has both a functional and a structural role at the synapse. Indeed, PRRT2 participates in the regulation of neurotransmitter release, as well as of actin cytoskeleton dynamics during synaptogenesis. Here, we show a role of the protein also during early stages of neuronal development. We found that PRRT2 accumulates at the growth cone in cultured hippocampal neurons. Overexpression of the protein causes an increase in the size and the morphological complexity of growth cones. In contrast, the growth cones of neurons derived from PRRT2 KO mice are smaller and less elaborated. Finally, we demonstrated that the aberrant shape of PRRT2 KO growth cones is associated with a selective alteration of the growth cone actin cytoskeleton. Our data support a key role of PRRT2 in the regulation of growth cone morphology during neuronal development.

## 1. Introduction

Mutations in the PRRT2 gene cause a broad spectrum of paroxysmal neurological diseases including paroxysmal kinesigenic dyskinesia, episodic ataxia, benign familial infantile seizures, and hemiplegic migraine [1]. Biallelic loss of PRRT2 results in more severe phenotypes with developmental delay and cognitive difficulties [2], suggesting a functional role of the protein during neurodevelopmental events. In line with this, expression of PRRT2 was found to be altered in a fibroblast transcriptome analysis of a cohort of patients affected by schizophrenia, a well-known neurodevelopmental disorder [3]. In 2016, Liu and colleagues highlighted a delay in the migration of post-mitotic cortical neurons silenced for PRRT2 during embryogenesis [4]. More recently, PRRT2 was found among the proteins enriched in axonal growth cones of developing cortical neurons [5].

In mature neurons PRRT2 is enriched at synapses, where it participates to the regulation of neurotransmitter release by interacting with proteins of the synaptic vesicle fusion machinery [6]. Recently, we described a structural role for PRRT2, involving a link with the dynamic changes in the actin cytoskeleton which occur during the process of synaptogenesis [7]. These findings raise the possibility that PRRT2 plays a role also in the early stages of neuronal development, in which actin dynamics play a critical role.

Actin remodelling is a key process of the very first events of neuronal development [8], including polarisation, neuronal migration, and growth cone pathfinding, having a critical role in guiding axons to their final targets. The motile behaviour of growth cones and the protrusion of their leading-edge membranes are influenced by the interaction with extracellular matrix components and guidance cues that, through the activation of several actin-related pathways, modulate growth cone cytoskeleton [9]. Attractive stimuli induce the formation of protrusive elements at the growth cone plasma membrane (filopodia and lamellipodia) that sense the environment to parse the guidance information. These sensory structures are sites for adhesive engagement where actin filaments connect the local cytoskeleton to the surrounding surface. These focal contacts further stabilise the protrusions and, through the activity of actomyosin, convert the mechanical forces generated into movement towards the axonal target. On the other hand, repulsive cues cause the dissolution of actin filaments, ultimately leading to growth cone collapse and turning [10].

Here, we show that PRRT2 accumulates at the growth cone of in vitro developing hippocampal neurons and that the manipulation of its expression alters growth cone morphology. PRRT2 overexpressing growth cones are bigger and present more protrusions. In contrast, PRRT2 KO growth cones are smaller and more collapsed, probably due to a local imbalance in the expression of actin and adhesion proteins. Our data point to a novel function of PRRT2 in regulating the shape and function of neuronal growth cones. Dysregulation of this activity might contribute to the pathological features associated with human mutations in the PRRT2 gene.

## 2. Materials and Methods

### 2.1. Animals

Wild-type (WT) C57BL/6N mice were imported from Charles River (Calco, Italy). Knock-out (KO or PRRT2-/-) mice on C57BL/6N background were kindly provided by Prof. Benfenati (University of Genova, Genova, Italy). Mice were housed under constant temperature (22 ± 1 °C) and humidity (50%) conditions with a 12 h light/dark cycle and were provided with food and water ad libitum. All experiments involving animals followed protocols in accordance with the guidelines established by the European Communities Council (Directive 2010/63/EU of 4 March 2014) and were approved by the Institutional Animal Care and Use Committee (IACUC, permission number 796) of the San Raffaele Scientific Institute and the Italian Ministry of Health. All efforts were made to minimise animal suffering.

### 2.2. Plasmids

pCAG-IRES-tdTomato was kindly gifted by Dr. Laura Cancedda (Italian Institute of Technology, Genoa, Italy) [11]. Mouse Prrt2 cDNA was inserted into the pCAG-IRES-tdTomato through SacI/SmaI digestion of the vector and SacI/BamHI digestion of the donor. All primers were purchased from Sigma-Aldrich (St. Louis, MO, USA), and all restriction enzymes were from NEB.

### 2.3. Cell Culture Procedures

Primary neuronal cultures were prepared from the hippocampi of embryonic day 17.5 embryos from WT and PRRT2 KO mice of either sex, as previously described [12]. For low-density cultures, neurons were plated on poly-l-lysine (PLL; 0.2 mg/mL; Sigma #P2636)-coated 24 mm glass coverslips at a density of 120,000 cells per coverslip, and maintained as a sandwich co-culture with astroglia in modified Eagle’s medium (MEM Invitrogen, Carlsbad, CA, USA) supplemented with 1% N2 supplement (Gibco #1502-048), 2 mM l-glutamine, 1 mM sodium pyruvate (Sigma #P2256), and 4 mM glucose at 37 °C in 5% CO_2_ humidified atmosphere [13]. When indicated, PLL-coated coverslips were subsequently coated with laminin (LN; 20 μg/mL; Sigma #L2020).

### 2.4. Neuronal Cell Electroporation

After mechanical dissociation of the E17.5 hippocampi, hippocampal neurons in suspension were electroporated with the Basic Primary Neuron Nucleofector Kit (#VPI1003, Lonza, Basel, Switzerland). Briefly, 80 μL of solution 1 and 20 μL of solution 2 were mixed for each electroporation reaction. For each reaction, one million neuronal cells were pelleted at 1500 rpm for 5 min at room temperature (RT). The cell pellet was resuspended in 100 μL of electroporation mix. A total of 3 μg of DNA was added, and the cell suspension was transferred to an electroporation cuvette. Electroporation was performed with the O-05 program of the Amaxa Nucleofector Device (Amaxa Biosystems, Cologne, Germany). Then, 1 mL of fresh plating medium (MEM supplemented with 3.3 mM glucose, 10% horse serum (Gibco #26050), and 2 mM l-glutamine) was added, and cells were resuspended with a sterile Pasteur pipette provided with the kit and plated on PLL-coated coverslips. After 4–5 h, coverslips were turned upside down on glial cells.

### 2.5. Cell Labelling Protocols, Image Acquisition and Analysis

Immunofluorescence experiments were performed as previously described [14]. Briefly, cells were rinsed once with Krebs–Ringer solution (KRH)-EGTA (in mM: 130 NaCl, 5 KCl, 1.2 KH_2_PO_4_, 1.2 MgSO_4_, 2 MgCl_2_, 2 EGTA, 25 HEPES, and 6 glucose; pH 7.4) for neurons. Cells were fixed for 15 min with 4% paraformaldehyde (Sigma #441244), 4% sucrose in 120 mM sodium phosphate buffer (pH 7.4), supplemented with 4 mM EGTA. Coverslips were rinsed three times with phosphate-buffered saline (PBS) and then incubated at RT for 2 h or overnight at 4 °C in a humidified chamber with the primary antibodies appropriately diluted in goat serum dilution buffer (GSDB; 15% goat serum, 450 mM NaCl, 0.3% Triton X-100, and 20 mM sodium phosphate buffer; pH 7.4). Specimens were then washed three times with PBS and incubated with the appropriate secondary antibodies diluted in GSDB at RT for 1 h. Alexa Fluor 488/564-conjugated phalloidin (ThermoFisher, Waltham, MA, USA #A12379 and #A22283) was incubated together with the secondary antibodies (diluted 1:300) when indicated. After three washes with PBS, coverslips were mounted with Dako fluorescence mounting medium (Dako, Carpinteria, CA, USA # S3023). The following primary antibodies were used: anti-PRRT2 (kindly provided by Dr. Tsai; rabbit) 1:100, anti-tubulin beta III (Biolegend #801201; mouse) 1:1000. The following secondary antibodies were used: FITC goat anti-rabbit (Jackson Immuno Research, West Grove, PA, USA #111-095-003) 1:50, Alexa Fluor 405 goat anti-mouse (#A31553, Life Technologies – ThermoFisher, Waltham, MA, USA) 1:100. When indicated, nuclei staining was performed by incubating coverslips with the Hoechst 33342 dye (ThermoFisher) diluted 1:10,000 in PBS for 5 min during the last round of washes. Epifluorescence images were acquired with a Zeiss Axio Imager.A2 equipped with an AxioCam MRm camera, with 63× objective. Confocal images were acquired with a Leica TSC SP8 confocal microscopy, with a 63× objective. Image analysis was performed with the ImageJ software. Neurite length was measured with the NeuronJ plugin. Only neurites longer than the nucleus were considered. The actin protrusion area was measured as previously described [14]. Briefly, the protruding area was calculated by subtracting the area of βIII-tubulin mask (obtained by thresholding the βIII-tubulin channel) from the phalloidin mask. Growth cone morphology parameters were obtained using area, perimeter, and shape descriptors measurements of ImageJ.

### 2.6. Growth Cone Fractionation

Growth cone fractions were obtained as previously described [5]. Briefly, brains of P2/P3 mouse pups were rapidly homogenised in 0.32 M sucrose buffer supplemented with 4 mM HEPES and protease/phosphatase inhibitors (Sigma #P8340, #P2850, #P0044) with 13 strokes at 900 rpm in a glass-Teflon potter. Post-nuclear homogenates (input) were obtained as supernatants after centrifugation at 1700× *g* for 15 min. Inputs were layered onto 0.83 M sucrose and a 2.5 M sucrose cushion and centrifuged at 250,000× *g* for 50 min at 4 °C (Optima L-90K, SW55Ti rotor, Beckman Coulter, Brea, CA, USA). The growth cone fraction was extracted from the 0.32–0.83 M interface, while the non-growth cone fraction was extracted from the 0.83–2.5 M interface. Both fractions were then concentrated by centrifugation at 100,000× *g* for 1 h at 37 °C (TLX ultra, 120.2 rotor, Beckman Coulter). The pellet obtained was then resuspended with 1X Laemmli buffer (LB; 20 mM Tris-HCl (pH 6.8), 2 mM EDTA, 2% SDS, 10% glycerol, 2% β-mercaptoethanol, and 0.01% bromophenol blue).

### 2.7. SDS-PAGE and Western Blotting

Equal volumes of protein samples were subjected to SDS–polyacrylamide gel electrophoresis (SDS-PAGE) using a standard vertical gel electrophoresis unit (Hoefer, San Francisco, CA, USA). Samples were loaded after heating at 90 °C for 5 min. Markers were used as standards to extrapolate the molecular weight of the analysed protein samples (Biorad, Hercules, CA, USA; SDS-PAGE standard high range #1610303, SDS-PAGE standard low range #1610304, precision plus protein kaleidoscope #1610375). Electrophoretic separation was performed in running buffer 1X (50 mM Tris-HCl, 384 mM glycine, 0.1% SDS) at a voltage of 80 V in the stacking gel and 150 V in the running gel. The separated proteins were transferred to a nitrocellulose membrane (0.45 μm; GE Healthcare, Buckinghamshire, UK, #10600002). Transfer was performed overnight in transfer buffer (25 mM Tris-HCl, 192 mM glycine, 20% methanol) at 250 mA at 4 °C. The nitrocellulose membrane was stained with Ponceau Red (Sigma #P7170) to assess proper transfer. The membrane was cut horizontally in correspondence of the appropriate molecular weights and used for western blotting. Blocking of the membranes was performed in 5% milk in TBST (Tris-Base saline-Tween: 150 mM NaCl, 20 mM Tris-HCl (pH 7.4), 0.05% Tween 20) for 1 h at RT. Primary antibodies were appropriately diluted in 5% milk in TBST and incubated for either 2 h at RT or overnight at 4 °C. Membranes were washed three times in TBST to eliminate primary antibody in excess. Secondary horseradish peroxidase (HRP) conjugated antibodies were diluted 1:10,000 in 5% milk in TBST and incubated for 1 h at RT. Membranes were washed three times in TBST to remove secondary antibodies in excess. Detection was performed with the enhanced chemiluminescence reaction (ECL Prime; GE Healthcare #16929851). Signals were detected with Chemidoc (Biorad Chemidoc MP Imaging system, Hercules, CA, USA). Densitometric quantification was performed with ImageLab software (Biorad), and data analysis was performed with Excel (Microsoft, Redmond, WA, USA). The extent of phosphorylation of a protein of interest was analysed by loading the same sample on two separate gels run in parallel and considering the ratio between the phosphorylated and total forms normalised on their own GAPDH. The following primary antibodies were used: anti-Actin (Sigma #A5441, mouse; 1:1000); anti-PRRT2 (Sigma #HPA014447, rabbit; 1:1000); anti-GAPDH (Cell Signaling, Danvers, MA, USA, #2118, rabbit; 1:10,000); anti-pTyr416SRC (Cell signaling #2101, rabbit; 1:1000); anti-SRC (Cell Signaling #2108, rabbit; 1:1000); anti-pTyr397FAK (ThermoFisher #44-6246, rabbit; 1:1000); anti-β1-Integrin (homemade, kindly provided by Prof. de Curtis [15], rabbit; 1:1000); anti-NCAM (Millipore #AB5023, rabbit; 1:1000); anti-pan-cadherin (Sigma, #SAB4200731, mouse, 1:1000); anti-GluR1 (Cell Signalling, #13185, rabbit, 1:1000).

### 2.8. Statistical Analysis

Data were analysed using Microsoft Excel and GraphPad Prism 8.0 (La Jolla, CA, USA). Normal distribution of data was evaluated using the D’Agostino–Pearson normality test. In the case of normally distributed data, statistical comparisons were performed with two-sided Student’s *t*-test. When data distribution was not normal, the Mann–Whitney *U*-test was used. Sample size for each experiment was calculated using G*Power ver. 3.1 software (Heinrich-Heine-University, Dusseldorf, Germany) on the basis of effect sizes calculated from our preliminary or previously published data (appropriately referred to in the References section), with a power of 0.8 at the alpha = 0.05 level.

## 3. Results

### 3.1. PRRT2 Was Expressed in the Growth Cones of Developing Neurons

**Figure 1:**
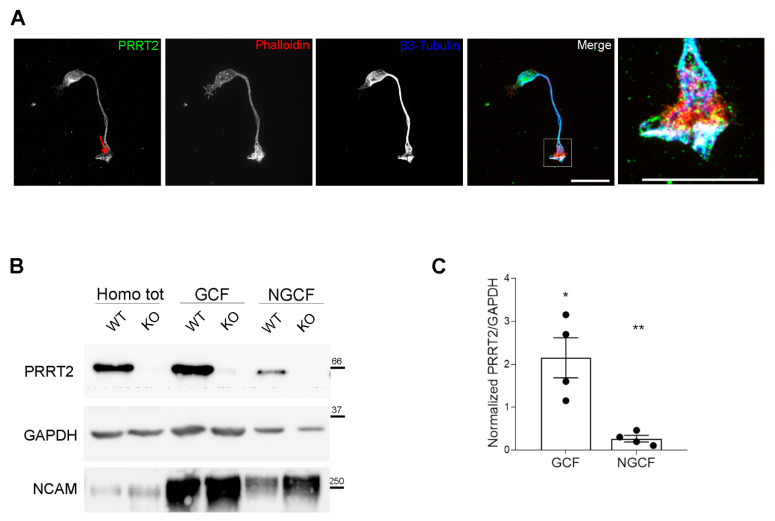
PRRT2 is enriched in the growth cones of in vitro developing neurons. (**A**) 1 DIV hippocampal neurons processed for immunofluorescence and stained for endogenous PRRT2, actin, and βIII-tubulin. Red arrow points to PRRT2 signal at the growth cone. The panel on the right shows a higher magnification of the inset in the Merge panel. Note the white signal at the tip of the growth cone, indicating overlapping of the signals for PRRT2, actin, and βIII-tubulin. Scale bar = 20 μm, inset scale bar = 10 μm. (**B**) Representative immunoblot of PRRT2 and NCAM in post-nuclear supernatant fraction (Homo tot), growth cone (GCF), and non-growth cone (NGCF) fractions from WT and PRRT2 KO P2/P3 brains. (**C**) Quantification of PRRT2 enrichment in WT fractions. PRRT2 band intensity was normalised on GAPDH for each sample and on the homogenate level, which was set to 1. Data are expressed as mean ± SEM of *n* = 4 embryos (Homo tot: 1, GCF: 2.2 ± 0.9, NGCF: 0.3 ± 0.1). One-way ANOVA + Tukey’s test; * *p* < 0.05 homo tot vs. GCF, ** *p* < 0.01 GCF vs. NGCF.

We wanted to address the subcellular localisation of PRRT2 in neurons at early stages of development. In particular, we were interested in understanding whether PRRT2 is expressed at the level of the growth cone, as suggested by proteomic analysis of growth cone fractions [5]. As depicted by the immunofluorescence shown in Figure 1A, PRRT2 is widely distributed in neurons at early stages of development, being detectable in the cell body, along the neurite, and at the growth cone of hippocampal neurons at 1 day in vitro (DIV), confirming the presence of the protein in this subcellular region. Through biochemical enrichment of growth cones from P2/P3 mouse brains, we found that the PRRT2 protein is enriched in the growth cone fraction, as compared to post-nuclear supernatant and non-growth cone fraction (Figure 1B,C).

### 3.2. PRRT2 Overexpression In Vitro Increased Growth Cone Complexity

**Figure 2:**
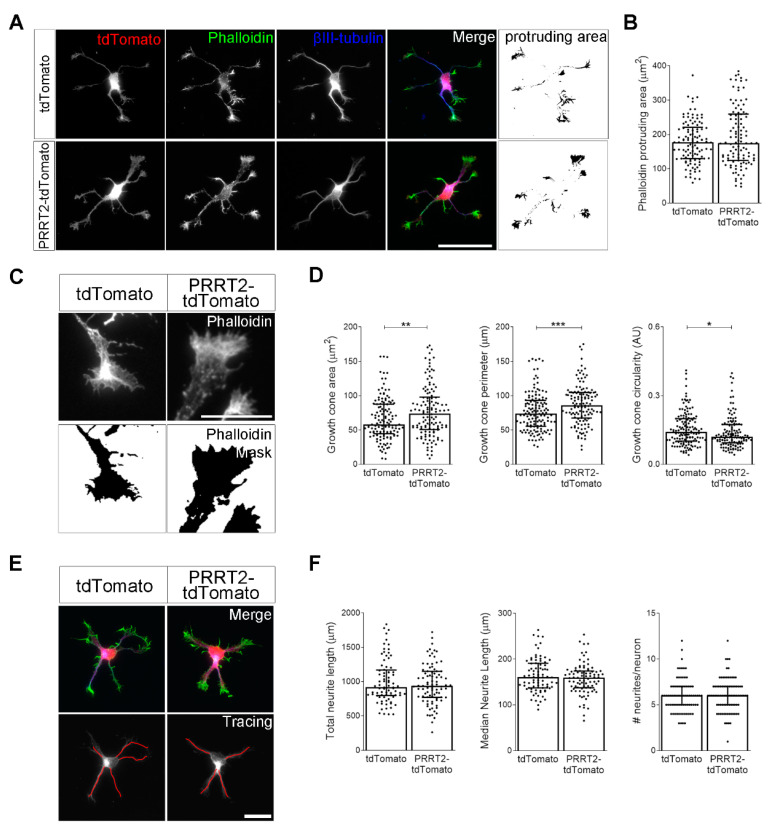
PRRT2 overexpression in developing hippocampal neurons increased growth cone dimension and complexity. (**A**) 1 DIV embryonic hippocampal neurons electroporated at 0 DIV with tdTomato or PRRT2-IRES-tdTomato. Neurons were labelled with phalloidin to stain F-actin and an anti-βIII-tubulin antibody for neuronal microtubules. Actin protrusion area was calculated by subtracting the area of βIII-tubulin signal from F-actin area (protruding area). Scale bar = 50 µm. (**B**) Quantitative evaluation of actin protrusion area of neurons in (**A**). Data are expressed as median values ± interquartile range of *n* = total number of neurons from three independent cultures (tdTomato = 110, PRRT2-tdTomato = 106). Median F-actin protruding area (µm^2^): tdTomato = 177.4, PRRT2-tdTomato = 174.9. Statistical significance was determined by Mann–Whitney test. (**C**) The biggest growth cone was selected from the mask of the phalloidin channel of neurons described in (**A**), and its area, perimeter, and circularity were measured. Scale bar = 15 µm. (**D**) Quantitative analysis of growth cone parameters. Data are expressed as median ± interquartile range of *n* = total number of growth cones from three independent cultures (tdTomato = 137, PRRT2-tdTomato = 126). Median growth cone area (µm^2^): tdTomato = 58.24, PRRT2-tdTomato = 74.20; median growth cone perimeter (µm): tdTomato = 73.96, PRRT2-tdTomato = 85.98; median growth cone circularity (A.U.): tdTomato = 0.142, PRRT2-tdTomato = 0.121. Statistical significance was determined by Mann–Whitney test; * *p* < 0.05, ** *p* < 0.01, *** *p* < 0.001. (**E**) The NeuronJ plugin of the ImageJ software was used to trace the neurites on the βIII-tubulin channel of neurons described in (**A**). Scale bars = 20 µm. (**F**) Quantitative analysis of neuritic elongation expressed as median neurite length, total neurite length, and number of neurites/neurons. Data are expressed as median ± interquartile range of *n* = total number of neurons from three independent cultures (tdTomato = 80, PRRT2-tdTomato = 86). Median neurite length (µm): tdTomato = 160.7, PRRT2-tdTomato = 159.3; median total neurite length (µm): tdTomato = 915.5, PRRT2-tdTomato = 937.3; median neurites number/neuron: tdTomato = 6, PRRT2-tdTomato = 6. Statistical significance was determined by Mann–Whitney test.

To evaluate whether the manipulation of PRRT2 expression could alter early neuronal development, we electroporated either PRRT2-IRES-tdTomato or tdTomato (control) plasmids in WT embryonic hippocampal neurons immediately before their seeding (0 DIV). After one day (1 DIV), neurons were processed for immunofluorescence with phalloidin to stain F-actin and anti-βIII-tubulin antibodies to mark microtubules. We measured the F-actin protruding area on the entire cell by subtracting the area of βIII-tubulin signal from the F-actin area (Figure 2A,B). We did not observe a perturbation of the global actin protruding area at 1 DIV in neurons overexpressing PRRT2.

To better focus on the effects of PRRT2 overexpression on growth cone morphology, we created a mask of the phalloidin channel and analysed the area, perimeter, and circularity of the major growth cone. Notably, we observed that PRRT2-overexpressing neurons presented a significant increase in both growth cone area and perimeter, as compared to control neurons (Figure 2C,D). Moreover, growth cones of PRRT2-overexpressing neurons had a circularity value slightly lower than control neurons, indicating that they were characterised by a more complex morphology.

Finally, we wanted to assess whether PRRT2 overexpression could affect neurite elongation of developing neurons. To this end, we measured the number and length of neurites of 1 DIV hippocampal neurons expressing tdTomato-PRRT2 or tdTomato (Figure 2E,F). We did not observe any significant variation of 1 DIV neuritic development. Together, these data suggest that PRRT2 overexpression specifically impacts on growth cone shape by increasing its dimension and complexity.

### 3.3. PRRT2 Knock-Out Altered the Complexity of Growth Cone Shape in Developing Neurons Plated on Laminin

**Figure 3:**
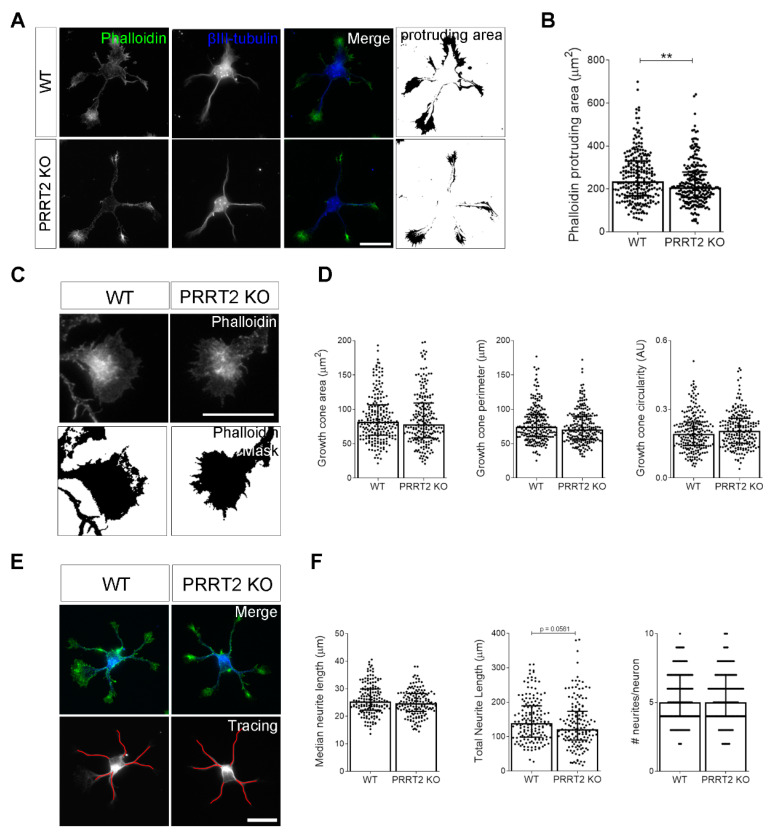
PRRT2 KO neurons display normal growth cone morphology. (**A**) Representative images of 1 DIV WT and PRRT2 KO hippocampal neurons. Neurons were labelled with phalloidin to stain F-actin and βIII-tubulin for neuronal microtubules. Scale bars = 20 µm. (**B**) Quantitative evaluation of actin protrusion areas. Actin protrusion area was calculated by subtracting the area of βIII-tubulin signal from F-actin area. Data are expressed as median values ± interquartile range of *n* = total number of neurons from four independent cultures (WT = 253, PRRT2 KO = 255). Median F-actin protruding area (µm^2^): WT = 235.5, PRRT2 KO = 207.8. Statistical significance was determined by Mann–Whitney test; ** *p* < 0.01. (**C**) The biggest growth cone was selected from the mask of the phalloidin channel on the neurons described in (**A**), and its area, perimeter, and circularity were measured. Scale bars = 20 µm. (**D**) Quantitative analysis of growth cone parameters. Data are expressed as median ± interquartile range of *n* = total number of growth cones from three independent cultures (WT = 223, PRRT2 KO = 220). Median growth cone area (µm^2^): WT = 81.87, PRRT2 KO = 77.86; median growth cone perimeter (µm): WT = 74.30, PRRT2 KO = 70.64; median growth cone circularity (A.U.): WT = 0.192, PRRT2 KO = 0.206. Statistical significance was determined by Mann–Whitney test. (**E**) The NeuronJ plugin of the ImageJ software was used to trace the neurites on the βIII-tubulin channel of neurons in (**A**). Scale bar = 20 μm. (**F**) Quantitative analysis of neuritic elongation expressed as median neurite length, total neurite length, and number of neurites/neurons. Data are expressed as median ± interquartile range of *n* = total number of neurons from three independent cultures (WT = 162; PRRT2 KO = 153). Median neurite length (µm): WT = 25.33, PRRT2 KO = 24.67; median total neurite length (µm): WT = 139.2, PRRT2 KO = 121.1; median neurites number/neuron: WT = 5, PRRT2 KO = 5. Statistical significance was determined by Mann–Whitney test.

We wanted to unravel whether depleting the activity of PRRT2 early during development could affect neuronal maturation. To this aim, we obtained hippocampal neurons from WT and PRRT2 KO embryos and fixed cells 1 day after plating. Neurons were processed for immunofluorescence and stained with phalloidin to decorate F-actin and with an antibody to βIII-tubulin to stain microtubules. We observed a slight, but statistically significant, reduction of the actin protruding area in PRRT2 KO neurons (Figure 3A,B). However, focusing on the morphological aspects of PRRT2 KO growth cones, we did not observe any significant difference in the area, perimeter, or circularity parameters of the major growth cone between WT and PRRT2 KO hippocampal neurons (Figure 3C,D). We tested the impact of PRRT2 loss on neurite elongation of developing neurons at 1 DIV and, also in this case, we did not find significant changes in neurite development in PRRT2 KO neurons compared to control cells (Figure 3E,F). These results suggest that PRRT2 deletion does not perturb the basal morphology of in vitro developing hippocampal neurons.

**Figure 4:**
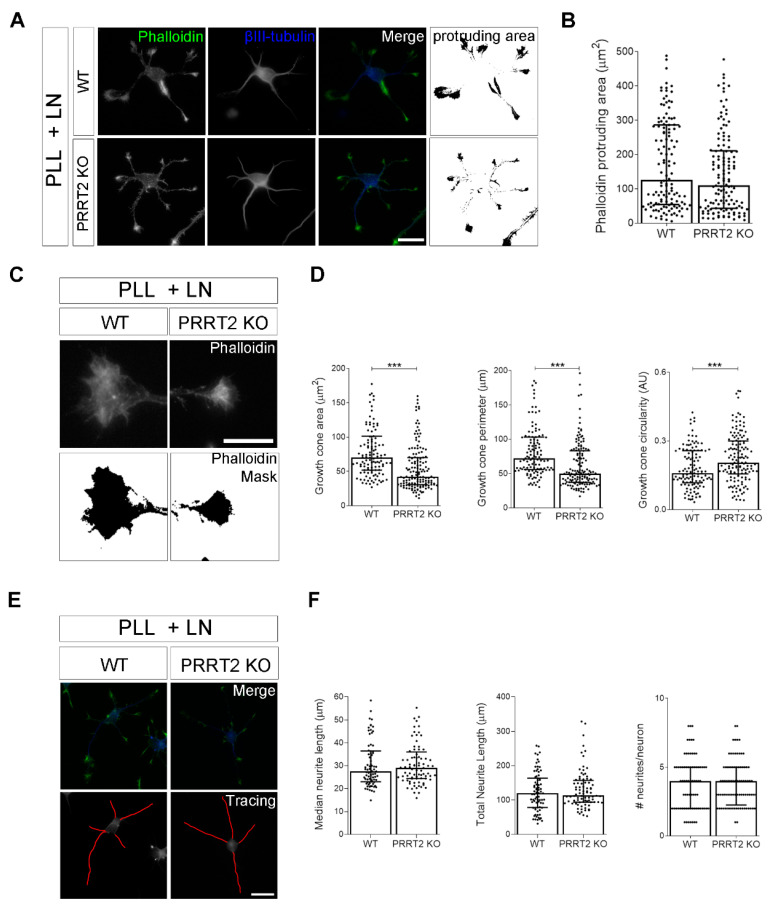
The loss of PRRT2 in hippocampal developing neurons plated on laminin disrupted growth cone size and complexity. (**A**) Representative images of 1 DIV WT and PRRT2 KO hippocampal neurons plated on poly-l-lysine and laminin (PLL + LN). Neurons were labelled with phalloidin to stain F-actin and βIII-tubulin for neuronal microtubules. Scale bars = 20 µm. (**B**) Quantitative evaluation of actin protrusion areas. Actin protrusion area was calculated by subtracting the area of βIII-tubulin signal from F-actin area. Data are expressed as median values ± interquartile range of *n* = total number of neurons from four independent cultures (WT = 130, PRRT2 KO = 134). Median F-actin protruding area (µm^2^): WT = 126.2, PRRT2 KO = 110.8. Statistical significance was determined by Mann–Whitney test. (**C**) The biggest growth cone was selected from the mask of the phalloidin channel on the neurons described in (**A**), and its area, perimeter, and circularity were measured. Scale bars = 20 µm. (**D**) Quantitative analysis of growth cone parameters. Data are expressed as median ± interquartile range of *n* = total number of growth cones from three independent cultures (WT = 119, PRRT2 KO = 142). Median growth cone area (µm^2^): WT = 70.35, PRRT2 KO = 42.47; median growth cone perimeter (µm): WT = 72.28, PRRT2 KO = 50.04; median growth cone circularity (A.U.): WT = 0.160, PRRT2 KO = 0.206. Statistical significance was determined by Mann–Whitney test; *** *p* < 0.001. (**E**) The NeuronJ plugin of the ImageJ software was used to trace the neurites on the βIII-tubulin channel of neurons in (**A**). Scale bar = 20 μm. (**F**) Quantitative analysis of neuritic elongation expressed as median neurite length, total neurite length, and number of neurites/neurons. Data are expressed as median ± interquartile range of *n* = total number of neurons from three independent cultures (WT = 78; PRRT2 KO = 84). Median neurite length (µm): WT = 25.33, PRRT2 KO = 24.67; median total neurite length (µm): WT = 119.9, PRRT2 KO = 114.4; median neurites number/neuron: WT = 4, PRRT2 KO = 4. Statistical significance was determined by Mann–Whitney test.

It is well known that the shape of growth cones is influenced by its interaction with the extracellular environment [16]. Thus, we measured the morphological parameters described above in PRRT2 KO neurons plated on laminin, a component of the extracellular matrix that represents a more physiological substrate with respect to poly-l-lysine. While under these conditions, the actin protruding area was unaffected by PRRT2 deletion (Figure 4A,B), the morphology of the growth cone was significantly altered. In particular, the area, perimeter, and circularity of the major growth cone of PRRT2 KO neurons were significantly reduced as compared to WT neurons (Figure 4C,D). Still, no significant differences were found in neurite development (Figure 4E,F). These data indicate that the loss of PRRT2 impacts growth cone morphological features in neurons grown on laminin.

### 3.4. The Actin Cytoskeleton Was Altered in PRRT2-Depleted Growth Cones

**Figure 5:**
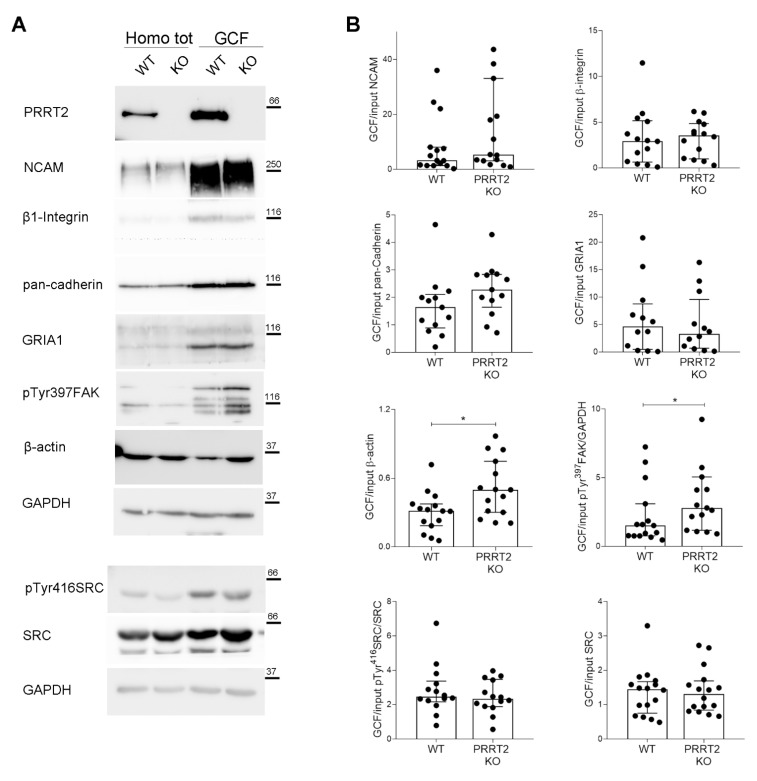
PRRT2 KO growth cones had an increased local actin and pTyr397FAK expression. (**A**) Representative immunoblot of PRRT2, NCAM, β1-Integrin, pan-cadherin, GRIA1, pTyr397FAK, β-actin, pTyr416SRC, SRC, and GAPDH in post-nuclear supernatant fraction (Homo tot) and growth cone fraction (GCF) from WT and PRRT2 KO embryonic brains. (**B**) Densitometric analysis of protein levels. Data are represented as ratio of GCF on total homogenate (Input). Median GCF/Input NCAM: WT = 3.2, PRRT2 KO = 5.4; β1-integrin: WT = 2.6, PRRT2 KO = 3.6; pan-cadherin: WT = 1.6, PRRT2 KO = 2.3; GluR1: WT = 4.6, PRRT2 KO = 3.2; β-actin: WT = 0.30, PRRT2 KO = 0.51, pTyr397FAK: WT = 1.5, PRRT2 KO = 2.8; pTyr416SRC: WT = 2.4, PRRT2 KO = 2.3; SRC: WT = 1.4, PRRT2 KO = 1.3. Data are expressed as median ± interquartile range of *n* = number of embryos. Mann–Whitney test; * *p* < 0.05.

To address whether the morphological defects found in PRRT2 KO growth cones were associated with an alteration of the local cytoskeleton, we analysed the expression of actin and actin-related proteins in the growth cones. To this aim, we isolated subcellular fractions enriched in growth cones from WT or PRRT2 KO mouse brains at postnatal stage P2-P3. We then performed Western blot analysis of actin and of proteins known to regulate the dynamics of the actin-based cytoskeleton in growth cones. In particular, we tested the cell adhesion molecule NCAM, β1-integrin and pan-cadherin receptors, the AMPA receptor subunit GRIA1, and the actin-regulating proteins SRC and FAK. As shown in Figure 5, actin was significantly more concentrated in growth cones from PRRT2 KO mice as compared to growth cones from WT mice. Moreover, we observed an increase in the phosphorylation state of FAK on Tyr 397, known to be responsible of initiating focal adhesion turnover [17]. No significant alteration was found on the relative levels of the other proteins. These results suggest that the morphological alterations observed in the growth cones of PRRT2 KO neurons were due to an unbalanced actin-related pathway, probably involving signals originating from the adhesive machinery of the growth cone.

## 4. Discussion

During brain development, the growth cone guides axonal navigation through the exploration of the local environment, detecting and responding to extrinsic cues to guide the direction of axonal extension. Cues involved in this guidance include chemotropic gradients and specific extracellular matrix substrates that, once interacting with their corresponding growth cone receptors, induce cytoskeletal reorganisation, ultimately leading to a modification of the shape and to a redirection of the extending growth cone [18]. In this study, we describe that PRRT2 accumulates at the growth cone of developing neurons, where it participates in the regulation of the local morphological changes in response to extracellular cues. We observe that in vitro hippocampal neurons express PRRT2 since the first stages of development. Moreover, through subcellular fractionation, we show that in vivo PRRT2 concentrates in the growth cone fraction from mouse brain.

Overexpression of PRRT2 causes an increase in the area, perimeter, and complexity of growth cones, pointing to an increase in the number and/or stability of protrusive elements at the membrane, such as lamellipodia and filopodia. This finding is in line with our previous observation that overexpression of PRRT2 in non-neuronal cell lines causes the appearance of actin-rich filopodia [7]. Growth cone filopodia are considered to be front-line guidance sensors at the tip of elongating axons during navigation and have a major role in establishing growth cone-adhesive substrate dynamic contacts during environmental exploration [9]. Indeed, adhesion complexes in the growth cone, known as point contacts, are mostly concentrated at the level of filopodia and share several components with canonical focal adhesions [19]. We also showed that overexpression of PRRT2 does not alter the general protrusiveness of the plasma membrane and neurite extension capability. Indeed, its effects are restricted to the growth cones, where PRRT2 is concentrated, indicating a specific effect and not a general alteration of cell activity and viability.

Interestingly, we found that PRRT2 KO developing neurons plated on an inert substrate, such as poly-L-lysine, did not show evident morphological abnormalities in either the general cell actin-protrusion area, growth cone size, and shape or neurite elongation parameters. However, when neurons were plated on laminin—one of the most expressed extracellular matrix components that targets specific receptors in the leading-edge membrane [20]—they exhibited important growth cone alterations. In particular, PRRT2 KO growth cones appeared smaller and rounder, resembling collapsed growth cones. Our results suggest that the defects of PRRT2 KO growth cones are attributable to a local alteration of the actin cytoskeleton. Indeed, we observed a statistically significant increase in the concentration of actin and pTyr397FAK in PRRT2 KO growth cones as compared to WT controls. The phosphorylation of FAK on tyrosine 397 promotes focal adhesion recycling [17], and it might explain the increased collapse of KO growth cones. This result agrees with our previous observation that PRRT2 overexpression in HeLa cells reduces the number of pTyr397FAK-positive focal adhesion [7].

Collectively, our results further support the idea of a key role of PRRT2 in conveying adhesive information to the local actin cytoskeleton in both developing and adult neurons. Mechanistically, it has been suggested that a lack of response to laminin-1 can be linked to either a downregulation of integrin receptors or to a decrease in their activation state [21]. We did not observe any significant alteration in the expression of β1-integrin and cadherin receptors at the growth cone level in PRRT2 KO brains. Therefore, a possible explanation may be that the lack of PRRT2 in growth cones causes an insensitivity to the adhesive signals generated by the binding of extracellular substrates, such as laminin, to their receptors. Through an as yet unidentified pathway, this may lead to an aberrant phosphorylation of FAK, which in turn regulates the polymerisation of the local actin cytoskeleton and ultimately influences growth cone shape and adhesion to the substrate. Further in vivo analyses will be needed to validate our observations in intact brain.

Most of the mutations affecting PRRT2 in patients induce a loss of function of the protein [22]. It is plausible to assume that the loss of PRRT2 function in neuronal growth cones during brain development might lead to circuit development alterations and miswiring, as suggested by results obtained in mice expressing pathogenic mutant forms of PRRT2 [4]. These abnormalities might contribute to the phenotypes observed in patients and could explain the more severe phenotypes that arise in biallelic-carrying patients.

## Data Availability

Not applicable.
